# The hypoxia marker CAIX is prognostic in the UK phase III VorteX-Biobank cohort: an important resource for translational research in soft tissue sarcoma

**DOI:** 10.1038/bjc.2017.430

**Published:** 2017-12-12

**Authors:** Laura Forker, Piers Gaunt, Stefano Sioletic, Patrick Shenjere, Robert Potter, Darren Roberts, Joely Irlam, Helen Valentine, David Hughes, Ana Hughes, Lucinda Billingham, Rob Grimer, Beatrice Seddon, Ananya Choudhury, Martin Robinson, Catharine M L West

**Affiliations:** 1 Translational Radiobiology Group, Division of Cancer Sciences, University of Manchester, Manchester Academic Health Science Centre, Christie Hospital NHS Foundation Trust, Wilmslow Road, Manchester M20 4BX, UK; 2 Cancer Research UK Clinical Trials Unit, Institute of Cancer and Genomic Sciences, University of Birmingham, Edgbaston, Birmingham B15 2TT, UK; 3 Department of Pathology, Ospedale S.Camillo de Lellis, Rieti 02100, Italy; 4 Department of Histopathology, The Christie NHS Foundation Trust, Wilmslow Road, Manchester M20 4BX, UK; 5 Department of Histopathology, Sheffield Teaching Hospitals NHS Trust, Weston Park Hospital, Whitham Road, Sheffield S10 2SJ, UK; 6 Department of Orthopaedic Oncology, Royal Orthopaedic Hospital NHS Foundation Trust, Bristol Road South, Northfield, Birmingham B31 2AP, UK; 7 Department of Oncology, University College London Hospitals NHS Foundation Trust, 1st Floor Central, 250 Euston Road, London NW1 2PG, UK; 8 Department of Oncology, Academic Unit of Clinical Oncology (Cancer Clinical Trials Centre), Weston Park Hospital, Whitham Road, Sheffield S10 2SJ, UK

**Keywords:** sarcoma, hypoxia, biomarker, CAIX, HIF-1*α*, GLUT1

## Abstract

**Background:**

Despite high metastasis rates, adjuvant/neoadjuvant systemic therapy for localised soft tissue sarcoma (STS) is not used routinely. Progress requires tailoring therapy to features of tumour biology, which need exploration in well-documented cohorts. Hypoxia has been linked to metastasis in STS and is targetable. This study evaluated hypoxia prognostic markers in the phase III adjuvant radiotherapy VorteX trial.

**Methods:**

Formalin-fixed paraffin-embedded tumour biopsies, fresh tumour/normal tissue and blood were collected before radiotherapy. Immunohistochemistry for HIF-1*α*, CAIX and GLUT1 was performed on tissue microarrays and assessed by two scorers (one pathologist). Prognostic analysis of disease-free survival (DFS) used Kaplan–Meier and Cox regression.

**Results:**

Biobank and outcome data were available for 203 out of 216 randomised patients. High CAIX expression was associated with worse DFS (hazard ratio 2.28, 95% confidence interval: 1.44–3.59, *P*<0.001). Hypoxia-inducible factor-1*α* and GLUT1 were not prognostic. Carbonic anhydrase IX remained prognostic in multivariable analysis.

**Conclusions:**

The VorteX-Biobank contains tissue with linked outcome data and is an important resource for research. This study confirms hypoxia is linked to poor prognosis in STS and suggests that CAIX may be the best known marker. However, overlap between single marker positivity was poor and future work will develop an STS hypoxia gene signature to account for tumour heterogeneity.

Soft tissue sarcomas (STS) are cancers of mesenchymal origin that are relatively rare, with ∼3000 cases per year diagnosed in adults in the United Kingdom (Francis *et al*, 2013). They display considerable heterogeneity; there are over 50 different malignant histologic subtypes (Fletcher *et al*, 2013) and can occur in any anatomical position (Clark *et al*, 2005). Most patients present with localised disease that can be managed with curative intent. A combination of surgery and radiotherapy results in high local control rates (Zagars *et al*, 2003). However, high-risk patients (high grade, deep, large tumours) have ∼50% 5-year survival (Weitz *et al*, 2003). Most deaths are due to distant metastasis. The use of chemotherapy in the adjuvant/neoadjuvant setting is controversial, due to lack of a consistent overall survival benefit in clinical trials (Pervaiz *et al*, 2008; Le Cesne *et al*, 2014). There is an important unmet clinical need to explore new strategies to prevent metastatic spread.

The failure of previous adjuvant/neoadjuvant trials may reflect the molecular heterogeneity of the disease. Progress requires identification of adverse features of tumour biology associated with metastasis that can be targeted with novel systemic agents. VorteX is a phase III randomised controlled trial assessing whether reduced adjuvant radiotherapy volume can improve limb function in adults with extremity STS. The VorteX-Biobank collected a range of tissue from this well-documented cohort and provides a resource to identify prognostic and predictive biomarkers for novel treatments.

Tumour hypoxia can drive metastasis (Sullivan and Graham, 2007) and has been associated with risk of distant metastasis in STS (Brizel *et al*, 1996). This is an attractive feature of tumour biology to manipulate, as it can be present across multiple subtypes despite molecular heterogeneity and there are multiple drugs in existence that can target hypoxic cell populations (Wilson and Hay, 2011). Some of these can reduce lung metastases in preclinical models in the adjuvant/neoadjuvant setting (Lunt *et al*, 2010; Liapis *et al*, 2015). A robust biomarker of tumour hypoxia in STS could potentially identify around half of patients within the current high-risk group who may benefit from hypoxia targeted therapy. Direct measurement of hypoxia using electrodes is impractical and imaging methods are unproven and not used in routine clinical practice (Hammond *et al*, 2014; Fleming *et al*, 2015). Previous studies exploring the link between endogenous hypoxia markers (proteins known to be expressed under hypoxia) have yielded conflicting results (Måseide *et al*, 2004; Shintani *et al*, 2006; Hoki *et al*, 2007; Huang *et al*, 2010; Smeland *et al*, 2012; Kim *et al*, 2015) and were limited by low numbers and old samples. This study evaluates the prognostic value of the endogenous markers HIF-1*α*, CAIX and GLUT1 in a large, modern (2013) phase III trial cohort treated with current surgical and radiotherapy techniques (VorteX-Biobank). REMARK guidelines for biomarker studies were followed (McShane *et al*, 2005) and manual *vs* automated scoring was compared.

## Materials and methods

### Patients and samples

Prospective samples were collected for the VorteX Biobank from consenting adult patients with localised, extremity soft tissue sarcoma receiving surgery with adjuvant radiotherapy as part of the phase III randomised controlled VorteX trial. The study had appropriate ethical approval (LREC 06/MRE/03/3) and informed consent was obtained for sample collection and analysis. Fresh tumour, matched normal tissue, formalin-fixed, paraffin-embedded (FFPE) tissue and peripheral blood samples were collected prior to radiotherapy.

### Construction of tissue microarrays

Tumour areas in FFPE material were demarcated by the VorteX trial histopathologist (DH) and 1 mm diameter cores were taken in triplicate from different areas. A maximum of 120 cores were placed within a single FFPE block in a standardised pattern (MTA-1; Beecher Instruments, Silver Spring, MD, USA). Eleven tissue microarrays (TMAs) were prepared in total.

### Immunohistochemistry

Sections were prepared in duplicate from each TMA for staining for a marker of interest and matched negative control. Positive controls included FFPE sections of hypoxic and normoxic cell pellets and tissue sections from other tumours that had shown high or low expression of the markers in previous experiments (Hunter *et al*, 2014). Tumour capillary staining was used as an additional internal control for GLUT1 staining.

Hypoxia-inducible factor-1*α* and CAIX staining was performed using the Bond-Max Automated staining system (Leica Biosystems, Milton Keynes, UK). Slides were dewaxed and rehydrated before antigen retrieval at pH 9.0 for 40 min at 100 °C. Three per cent hydrogen peroxide solution was used to block endogenous peroxidases. For HIF-1*α* the primary antibody was mouse monoclonal HIF-1*α* (BD Biosciences, Oxford, UK; 610959) (1 : 50 dilution) and the negative control was mouse IgG1 (Dako, Ely, UK; X0931). For CAIX the primary antibody was mouse monoclonal NCL-L-CAIX (Novacastra, Leica Biosystems, Milton Keynes, UK) (1 : 100 dilution) and the negative control was mouse IgG2a (Dako; X0943). All dilutions were in antibody diluent (Leica; AR9352) and negative controls were diluted to the same protein concentration as the primary. Slides were incubated for 8 min at room temperature with postprimary rabbit anti-mouse link reagent (Bond Polymer Refine Detection System; Leica; DS9800) and then for a further 8 min with anti-rabbit polymer-HRP detection reagent (Bond Polymer Refine Detection System; Leica). 3,3′-Diaminobenzidine tetrahydrochloride was applied for 10 min at room temperature. Slides were then counterstained with haematoxylin.

Glucose transporter 1 staining was performed manually. Slides were dewaxed and rehydrated. Three per cent hydrogen peroxide solution was used to block endogenous peroxidase activity and casein (Vector, Peterborough, UK; SP5020) was used as a protein block. Primary antibody (rabbit polyclonal anti-GLUT1; Alpha Diagnostic International, Source Bioscience, Nottingham, UK; GT-12A 10 μg ml^−1^) or negative control (rabbit IgG Vector I-1000 10 μg ml^−1^) was incubated with the slides for 1 h at 37 °C. Slides were then incubated with secondary antibody (Rabbit Envision Plus HRP Kit; Dako; K4010) for 30 min at room temperature. DAB+ (20 μl chromogen to 1 ml substrate) was applied for 5 min at room temperature. Slides were counterstained with haematoxylin for 1 min.

### Manual scoring of immunohistochemistry markers

Slides were viewed using Leica SCN400 Image Viewer and scored at × 8 magnification. The percentage of tumour cells per core expressing each marker was determined. Intensity was recorded for potential future use, but was not used in the current analysis in favour of a simpler scoring system. Negative controls were available for comparison. For HIF-1*α* only nuclear staining was considered, for CAIX only membrane staining was scored and for GLUT1 membrane and cytoplasmic staining were included.

Cores were scored twice by the same scorer (LF) on different days. For HIF-1*α* and GLUT1 all cores were also scored by a specialist sarcoma consultant histopathologist (SS) and for CAIX this was done for all cores with staining present (PS). Scorers were blinded to clinical outcomes. The score of the consultant histopathologist was taken if scores were discordant. The final score was an average across all cores for each tumour type. Definitions of marker positivity were selected to reflect those used in previous publications.

### Automated image analysis for scoring of immunohistochemistry markers

Tissue microarray images were imported into Definiens tissue studio v.4.2 (Definiens, Munich, Germany) and each core labelled with a coordinate according to location. Scoring algorithms were generated and iteratively improved for each marker with quantifiably expressed instructions for tissue detection, nuclear detection, cell stimulation and scoring thresholds (Supplementary Figure 1).

### Statistical analysis

The trial primary outcome measures were limb functionality and time to local recurrence, whereas secondary outcomes were disease-free survival (DFS) and overall survival (OS). The clinical outcome measure for the prognostic analysis was DFS, defined as the time from randomisation to local recurrence, metastasis or death. Patients without a DFS event were censored at the date last known to be alive and event free. Estimates were calculated using Kaplan–Meier (KM) analysis. The log-rank test was used to determine any difference in the outcome for each biomarker split combination. Hazard ratios (HRs) and 95% confidence intervals (CIs) were obtained using Cox regression analysis (univariable and multivariable). Univariable Cox modelling was used to identify clinicopathologic factors correlated to DFS in this cohort including grade, size (combined as stage), depth, gender, surgical margin, tumour location (upper or lower limb), WHO performance status and age. Factors significant at the 5% level in the univariable analysis were included in multivariable analyses with each of the study biomarkers. Spearman’s correlation and Bland–Altman (Bland and Altman, 1986) plots were used to assess intra- and interobserver variability for duplicate and independent scores per core and to compare manual and automated scores per core. Analysis was performed using Stata v.14 (College Station, TX, USA).

## Results

### Available material

**Figure 1:**
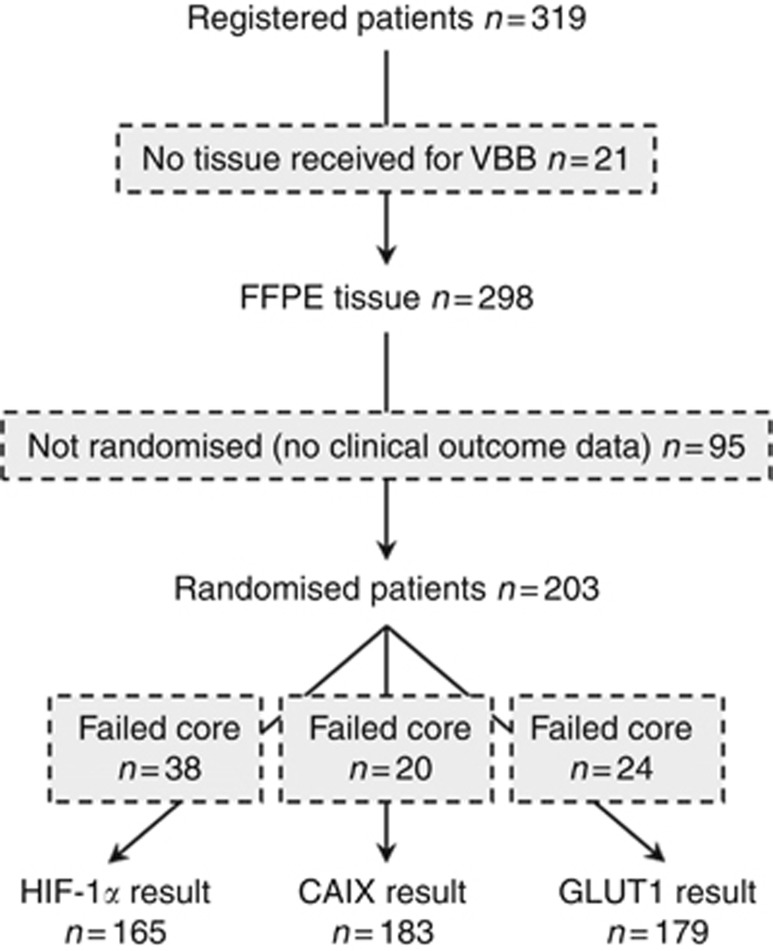
**CONSORT diagram.** Data for HIF-1*α*, CAIX and GLUT1 expression were available for 165, 183 and 179 patients randomised in the VorteX trial, respectively. CAIX=carbonic anhydrase IX; FFPE=formalin-fixed, paraffin-embedded; HIF-1*α*=hypoxia-inducible factor-1*α*; GLUT1=glucose transporter 1; VBB=VorteX-Biobank.

Between 2007 and 2013, 319 patients were registered. Of these, 216 patients met the eligibility criteria and were randomised between two different volumes of adjuvant radiotherapy. At the time of analysis median follow-up using reverse KM methodology was 5.2 years and event rates were 12% (25 out of 216) and 40% (86 out of 216) for local recurrence and any DFS event, respectively. Two hundred and three of 216 randomised patients (with complete clinical outcome data) consented to the VorteX-Biobank. Table 1 summarises the baseline characteristics in the Biobank population. Tissue collected is summarised in Supplementary Table 1. Staining results and clinical outcome data were available for 165, 183 and 179 patients for HIF-1*α*, CAIX and GLUT1, respectively (Figure 1). A scoring result was not available for every patient due to TMA degradation, lack of tumour in core or poor image quality.

### Protein marker expression

Supplementary Figure 2 shows staining patterns for the three markers. Hypoxia-inducible factor-1*α* staining was clearly nuclear and CAIX clearly membranous, consistent with the expected location for activity of these proteins. Very little additional staining was seen for these markers when compared to the negative control. GLUT1 staining was membranous and cytoplasmic, both were scored as a GLUT1 score based on both membranous and cytoplasmic staining has previously been shown to correlate with oxygen electrode measurements in cervix cancer (Airley *et al*, 2001). Results and thresholds defining positivity for each marker are included in Supplementary Table 2. The median of all scores was zero for all three markers; therefore, cores with any staining present were considered positive. The median of all results greater than zero was used to define strong positive. Duplicate and independent scores per core correlated well (Spearman’s *ρ*>0.8) and Bland–Altman analyses comparing scores are included in Supplementary Table and Supplementary Figure 5. There was poor overlap for positivity for markers within the same tumour sample (Supplementary Figure 4).

### Prognostic significance

Grade and size (combined as stage) and depth were significant in univariable analyses (Table 2) and incorporated in the multivariable analysis. Gender, surgical margin, tumour location (upper or lower limb), WHO performance status and age were not prognostic in the univariable analysis.

**Figure 2:**
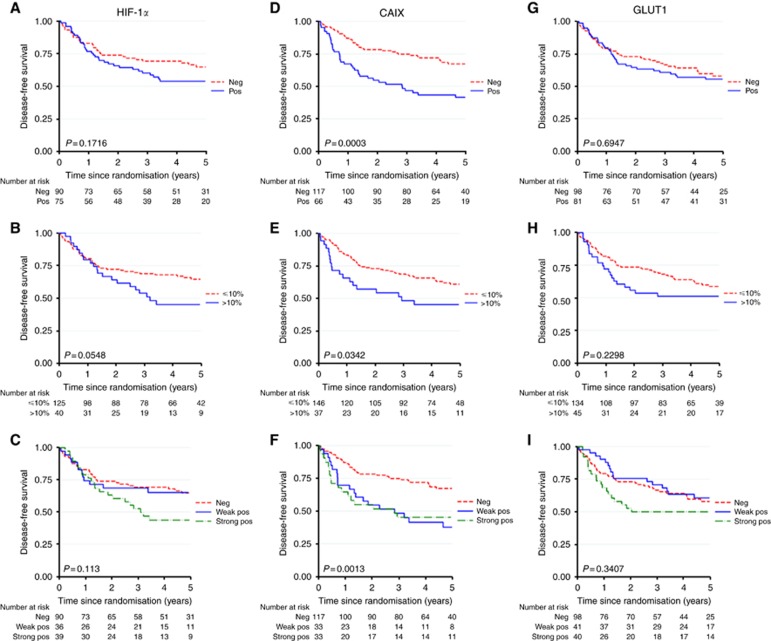
**Kaplan–Meier survival estimates for HIF-1*α* (A–C), CAIX (D–F) and GLUT1 (G–I).** (**A**) HIF-1*α*-positive *vs* –negative, (**B**) HIF-1*α* ⩽10% *vs* >10%, (**C**) HIF-1*α* strong positive, weak positive, negative, (**D**) CAIX positive *vs* negative, (**E**) CAIX ⩽10% *vs* >10%, (**F**) CAIX strong positive, weak positive, negative, (**G**) GLUT1 positive *vs* negative, (**H**) GLUT1 ⩽10% *vs* >10% and (**I**) GLUT1 strong positive, weak positive, negative.

Kaplan–Meier survival estimates for HIF-1*α*, CAIX and GLUT1 are presented in Figure 2. The *P*-value reported on the figures is from the log-rank test. When patients were split into two groups based on HIF-1*α* positivity (median split or >10% stained), there was no statistically significant difference in DFS (HR 1.41, 95% CI: 0.86, 2.31, *P*=0.174 for median split, HR 1.66, 95% CI: 0.98, 2.81, *P*=0.058 for >10% stained). When patients were split into three groups (strong positive, weak positive, negative) strong positive *vs* negative staining was associated with worse DFS (HR 1.76, 95% CI: 1.01, 3.07, *P*=0.048). This was not significant on multivariable analysis (HR 1.31, 95% CI: 0.74, 2.32, *P*=0.348).

Carbonic anhydrase IX was the strongest prognostic marker and was the only marker to retain prognostic significance in any of the multivariable analysis. Poor DFS was associated with CAIX values above the median (HR 2.28, 95% CI: 1.44, 3.59, *P*<0.001) or >10% stained (HR 1.75, 95% CI: 1.04, 2.94, *P*=0.037). These estimates remained significant for the median split in the multivariable analyses (HR 2.04, 95% CI: 1.29, 3.25, *P*=0.002), but not at the >10% stained split (HR 1.60, 95%CI: 0.95, 2.71, *P*=0.078). There was no observable difference between strong and weak positive values for CAIX with both being graphically comparable (Figure 2f). There was no difference in baseline characteristics between CAIX-positive and -negative patients (Supplementary Table 4).

Glucose transporter 1 was not prognostic (HR 1.10, 95% CI: 0.69, 1.73, *P*=0.695 – median split, HR 1.36, 95% CI: 0.82, 2.26, *P*=0.232 – >10% stained, HR 1.39, 95% CI: 0.80, 2.41, *P*=0.238 – strong positive *vs* negative).

### Comparison of manual and automated scoring

Most cores were successfully scored by automated analysis. Cores were excluded if there was insufficient tissue or core quality was poor. Correlation between manual and automated scores was relatively poor (Spearman’s *ρ*<0.72) and Bland–Altman analyses comparing scores are included in Supplementary Table and Supplementary Figure 5. KM survival estimates for HIF-1*α* and CAIX automated scores are shown in Supplementary Figure 6.

## Discussion

Owing to its rarity and heterogeneity, acquiring adequately sized cohorts of STS patients with both high-quality tumour tissue and robust clinical outcome data is extremely challenging. VorteX is the largest adjuvant radiotherapy trial completed in STS and provides a substantial cohort of patients with high-risk, localised disease covering a range of the more common subtypes seen in adults. The VorteX-Biobank was successful in collecting a wide range of tissue from these patients including FFPE tissue from 301 (94%) of enrolled patients and 203 (98%) of randomised patients and matched tumour and normal tissue from 190 (88%) of randomised patients. This has created a valuable resource for the study of STS biology and identification and validation of biomarkers for novel treatment strategies and highlights the importance of tissue banking alongside clinical trials whenever possible for rare cancers.

Staining patterns for endogenous immunohistochemistry markers of tumour hypoxia were consistent with previous studies in STS (Kim *et al*, 2015) and other cancers (Airley *et al*, 2003; Hunter *et al*, 2014). Spearman’s correlation for duplicate and independent scores was high. However, Bland–Altman analyses revealed that while the mean difference between scores was low between independent scores for all markers, the upper and lower limits of agreement were relatively high for CAIX (−28.9, 15.3) and GLUT1 (−19.7, 16.1) (Supplementary Figure 3). For CAIX this can be attributed to the fact that only positive cores were second scored for this marker, as due to the quality of the antibody and the staining pattern it was clear if a core was negative. As the optimal definition of positive for CAIX was the presence of any staining, a difference in scores between observers would not alter the classification of the sample if it was used as a biomarker. Although automated scoring would reduce analysis time and the need for specialist pathology input, this was not comparable to manual scoring by a sarcoma pathologist. Correlation between scores and Bland–Altman bias were not within acceptable limits (Supplementary Table and Supplementary Figure 5) and when KM analyses were performed for HIF-1*α* and CAIX using automated scores neither were found to be prognostic (Supplementary Figure 6). This is probably due to difficulty in using the software to define regions of stained tumour cells *vs* stroma or artefact.

Thresholds for marker positivity were selected to make results comparable with previously published studies. For HIF-1*α* this was difficult as previous studies have varied widely from considering >10% staining of any intensity as positive (Huang *et al*, 2010), to defining only tumours with over 50% of cells strongly stained as positive (Smeland *et al*, 2012). In the current work, intensity of staining was recorded for potential future use but was not considered at present as a simpler scoring system is likely to be more easily reproducible. For the CAIX studies evaluating membrane staining, one has used a cutoff value of 10% stained (Kim *et al*, 2015), and another has defined any staining as positive as it demonstrated no difference in outcome between patients classified as strongly or weakly positive (Måseide *et al*, 2004). This is consistent with the current work.

Hypoxia-inducible factor-1*α* and CAIX expression are associated with worse DFS, albeit CAIX was the only hypoxia marker that was significant in the multivariable analysis. Glucose transporter 1 was not prognostic. Four of five previous studies (Shintani *et al*, 2006; Hoki *et al*, 2007; Huang *et al*, 2010; Smeland *et al*, 2012; Kim *et al*, 2015) have associated high HIF-1*α* expression with adverse outcomes. Two of three previous studies (Måseide *et al*, 2004; Smeland *et al*, 2012; Kim *et al*, 2015) have associated high CAIX expression with poor outcomes in STS, and high GLUT1 expression has been shown to correlate with worse outcomes in one of two prior studies (Smeland *et al*, 2012; Kim *et al*, 2015). Results of the largest previous study (Smeland *et al*, 2012) (∼200 patients) were almost opposite to the current work with no association for HIF-1*α* and CAIX, but prognostic value reported for GLUT1. This may be explained by the fact that the previous study reported different staining patterns that would be considered unusual for the markers in question (mainly cytoplasmic for all markers) and included relatively old samples. The staining patterns scored in the current work are more consistent with the expected location of the active proteins and the cohort is superior to those used in previous studies, as this is a modern phase III trial population (2007–2013) with recent tissue collection and robust outcome data.

Although the current work did not find HIF-1*α* staining to be prognostic, it may potentially still be of interest. A recent meta-analysis of 16 studies (5 in STS) evaluating the prognostic significance of HIF-1*α* in STS and bone sarcomas found high expression was associated with higher rates of metastases, poor OS and poor DFS (Li *et al*, 2016). A consistent association with poor DFS was found in the STS subgroup analysis, although this only considered 136 patients in three studies. A limitation of HIF-1*α* is that it is more difficult to score and define positivity than for CAIX, which can simply be classed as positive in the presence of any membrane staining. Based on this work, CAIX appears to be the best current known hypoxia marker for use on pretreatment biopsies in STS.

Tissue microarrays rather than whole sections were used for this study due to the large number of samples. This is a potential limitation of the work as it assesses a smaller area than a whole section and therefore may be more likely to misclassify a hypoxic tumour as normoxic. However, this was assessed in one of the previous studies of CAIX in STS and TMAs were deemed accurate in determining marker expression provided that at least three cores were taken from two separate areas of tumour (Måseide *et al*, 2008). Another concern was that there was very limited overlap between expression of the markers in these clinical samples. All three markers are expressed under hypoxia and a degree of coexpression was expected. This may reflect the considerable molecular heterogeneity of STS and indicates that expression of these markers is likely influenced by other pathways. There is therefore a need for a more robust measure of hypoxia in STS than a single protein marker.

Hypoxia-related RNA expression signatures measure changes in multiple genes in response to hypoxia and can account for greater heterogeneity (Harris *et al*, 2015). Evaluation of a biomarker at the RNA level also reduces inconsistencies in methodology and interpretation between laboratories compared with IHC and is more likely to be reproducible in clinical practice. Hypoxia RNA expression signatures have made the most progress towards use as predictive biomarkers for hypoxia targeted therapy in the clinic, with two signatures (Toustrup *et al*, 2012; Eustace *et al*, 2013) currently undergoing prospective validation in phase III trials in head and neck cancer. Recently, one of these signatures has demonstrated prognostic value in a cohort of STS patients (Aggerholm-Pedersen *et al*, 2016). However, hypoxia signatures generally perform best in the tumour types they were developed for (Eustace *et al*, 2013) and the signature genes correlated poorly with oxygen electrode measurements in 16 patients indicating that it may not be a good measure of hypoxia in STS (Aggerholm-Pedersen *et al*, 2016). It may be more appropriate to derive a hypoxia signature specific to STS, as it must be a robust measure of hypoxia if it is to be used as a predictive biomarker for hypoxia targeted therapy. The other tissue available in the VorteX-Biobank is intended to be used for this purpose with the eventual aim of a biomarker-driven trial of hypoxia targeted adjuvant/neoadjuvant therapy in STS.

In summary, evaluation of protein hypoxia markers in a modern phase III clinical trial cohort suggests that CAIX is the best current known hypoxia marker. A simple binary scoring system can easily be implemented to define positivity but still requires manual pathology assessment. However, other measures of hypoxia such as gene signatures may perform better and be more reflective of hypoxia across different subtypes, compensating for greater heterogeneity. Further work is needed to develop a gene signature and confirm that it is a true measure of hypoxia by comparing this to CAIX and other methods of assessing hypoxia (e.g. imaging/electrodes) before this could be taken forward as a prognostic or predictive biomarker for clinical trials.

## Figures and Tables

**Table 1:** Baseline characteristics

	***n*****=203**	**%=100**
**Age**		
Median 60 years, interquartile range 48–69 years		
**Gender**		
Male	121	60
Female	82	40
**WHO performance status**		
0–1	163	80
2–3	5	2
Unknown	35	18
**Grade**		
1	14	7
2	39	19
3	150	74
**Depth**		
Superficial	36	18
Deep	166	82
Unknown	1	0
**Size**		
T1 (⩽5 cm)	19	9
T2 (>5 cm)	184	91
**Surgical margin**		
Intralesional	5	2
Marginal	79	39
Wide	119	59
**Histologic subtype**		
Dedifferentiated liposarcoma	5	2
Extraskeletal myxoid chrondrosarcoma	4	2
Leiomyosarcoma	9	4
Malignant peripheral nerve sheath tumour	6	3
Malignant solitary fibrous tumour	4	2
Myxofibrosarcoma	52	26
Myxoid liposarcoma	27	13
Pleomorphic liposarcoma	5	2
Pleomorphic rhabdomyosarcoma	3	1
Synovial sarcoma	6	3
Undifferentiated pleomorphic sarcoma	68	33
Other	13	6
Unknown	1	0

Abbreviation: WHO=World Health Organisation.

**Table 2:** Univariable analyses

	**HR (95% CI)**	***P*****-value**
**Age**		
	1.00 (0.99, 1.02)	0.616
**Gender**		
Female	1	—
Male	1.18 (0.76, 1.86)	0.460
**WHO performance status**		
0	1	
1	1.43 (0.83, 2.48)	0.202
2	1.37 (0.33, 5.65)	0.661
**Stage (grade, size)**		
I	0.46 (0.17, 1.26)	0.131
II	0.29 (0.15, 0.59)	0.001
III	1	—
**Depth**		
Deep	1	—
Superficial	0.46 (0.22, 0.95)	0.037
**Surgical margin**		
Wide	1	—
Intralesional	1.15 (0.28, 4.75)	0.846
Marginal	1.19 (0.77, 1.85)	0.439
**Location**		
Lower limb	1	—
Upper limb	0.96 (0.77, 1.85)	0.898
**HIF-1*α* (median split)**		
Negative	1	—
Positive	1.41 (0.86, 2.31)	0.174
**CAIX (median split)**		
Negative	1	—
Positive	2.28 (1.44, 3.59)	<0.001
**GLUT1 (median split)**		
Negative	1	—
Positive	1.10 (0.69, 1.73)	0.695

Abbreviations: CAIX=carbonic anhydrase IX; CI=confidence interval; HIF-1*α*=hypoxia-inducible factor-1*α*; HR=hazard ratio; GLUT1=glucose transporter 1.
