# Supplementary figures and images for: The hypoxia marker CAIX is prognostic in the UK phase III VorteX-Biobank cohort: an important resource for translational research in soft tissue sarcoma
Source: Br J Cancer. 2017 Dec 12;118(5):698–704. doi: 10.1038/bjc.2017.430 (PMC5846059; doi:10.1038/bjc.2017.430)

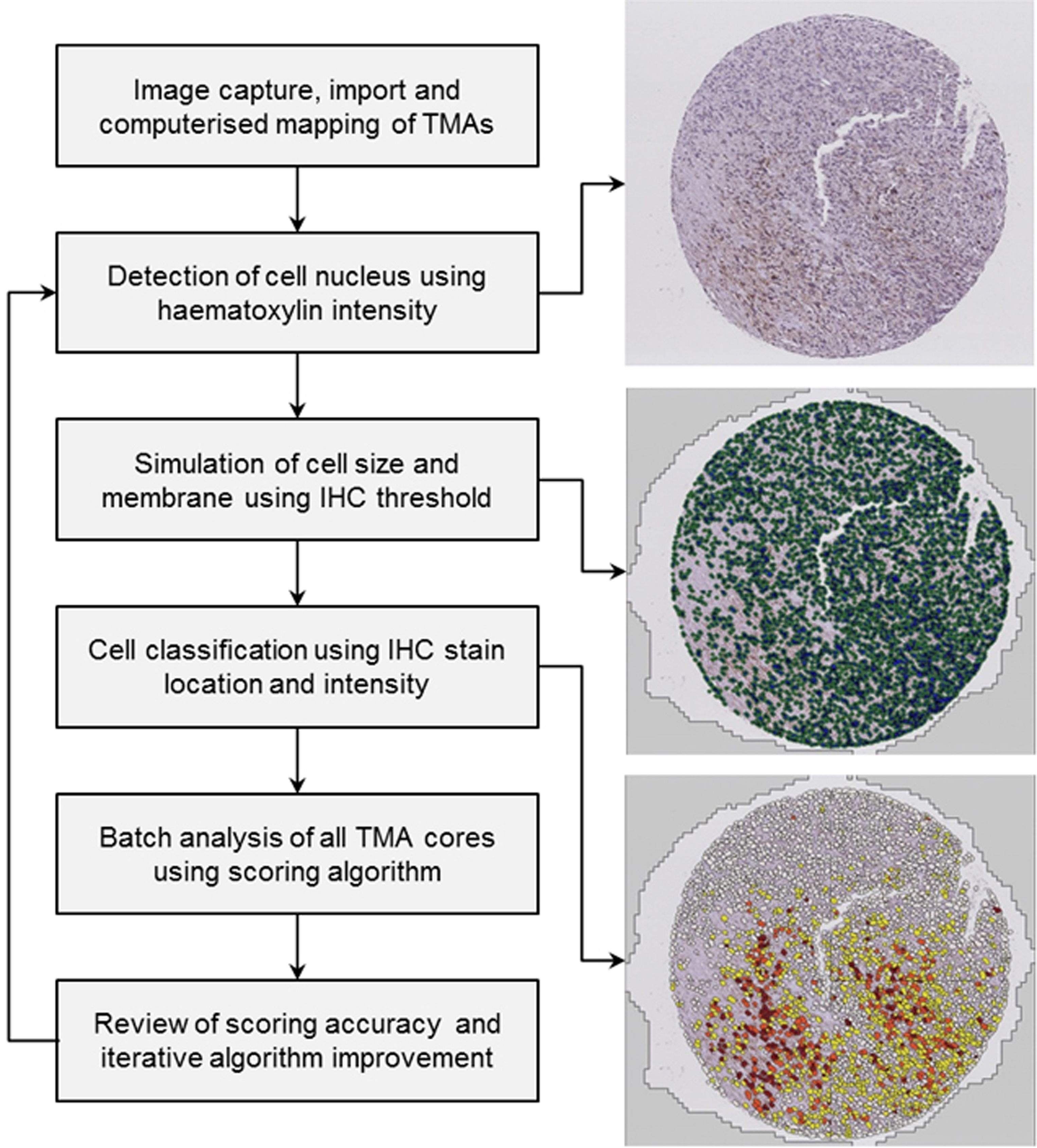

Supplement: Supplementary Figure 1 [file bjc2017430x1.tif]

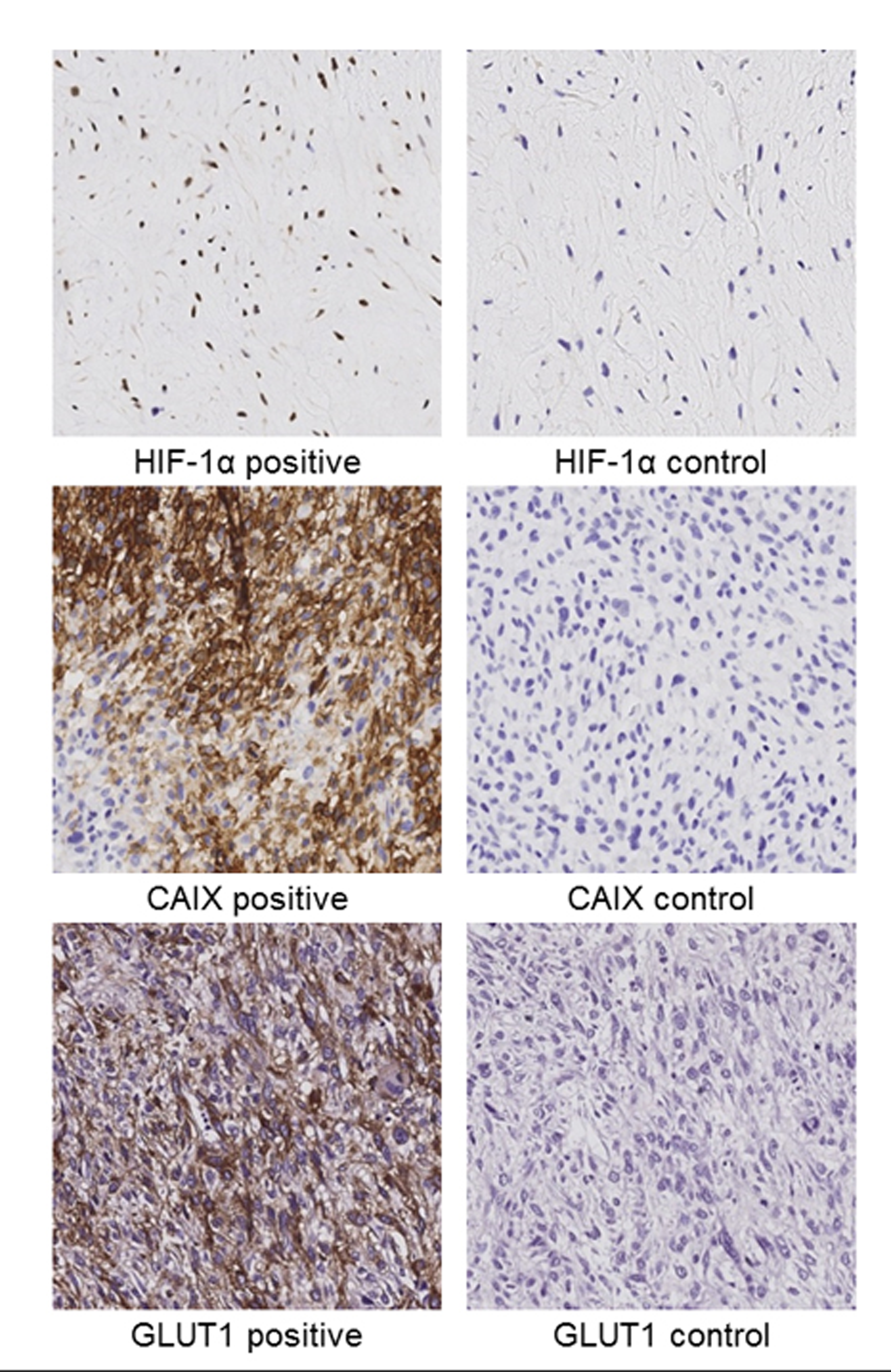

Supplement: Supplementary Figure 2 [file bjc2017430x2.tif]

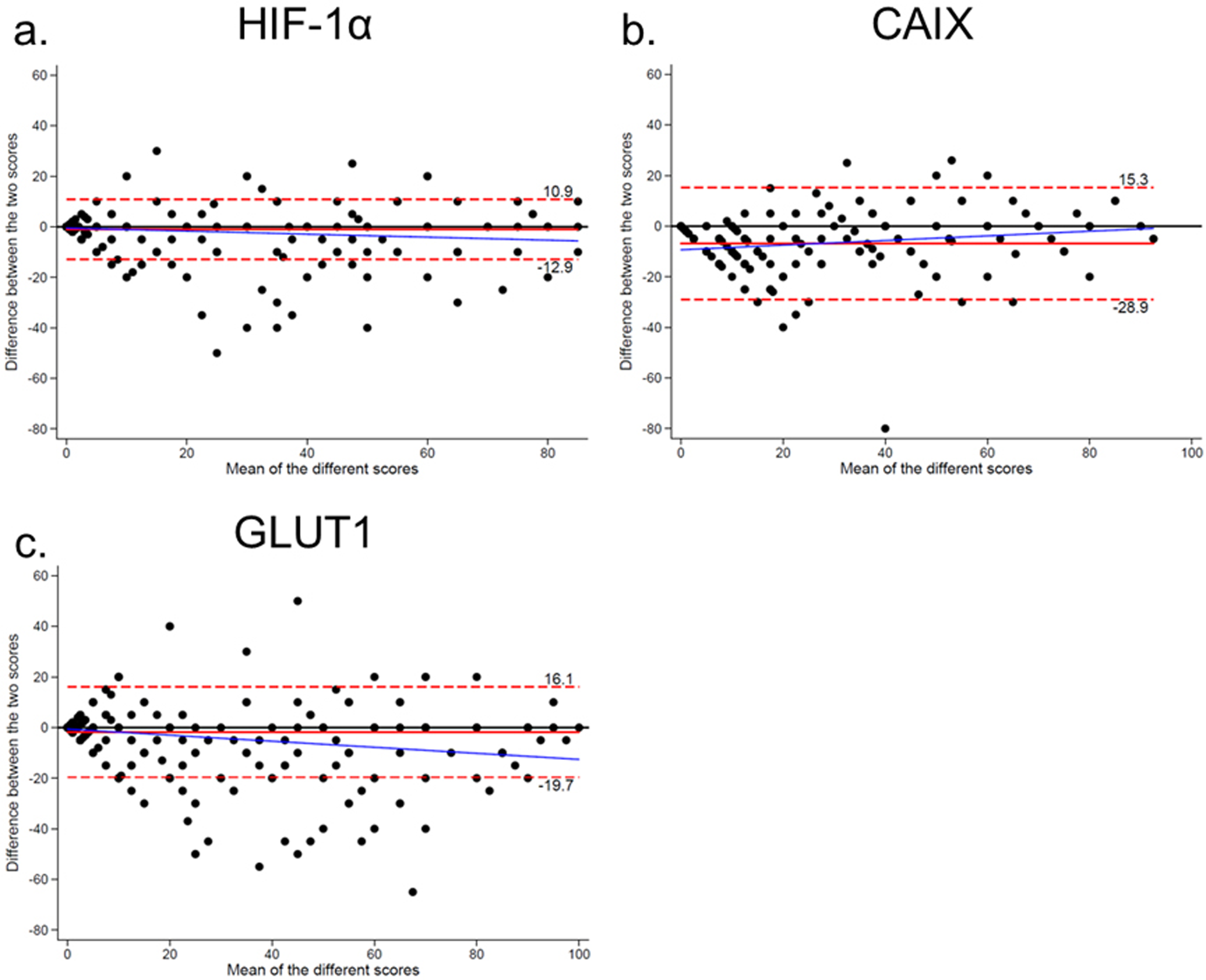

Supplement: Supplementary Figure 3 [file bjc2017430x3.tif]

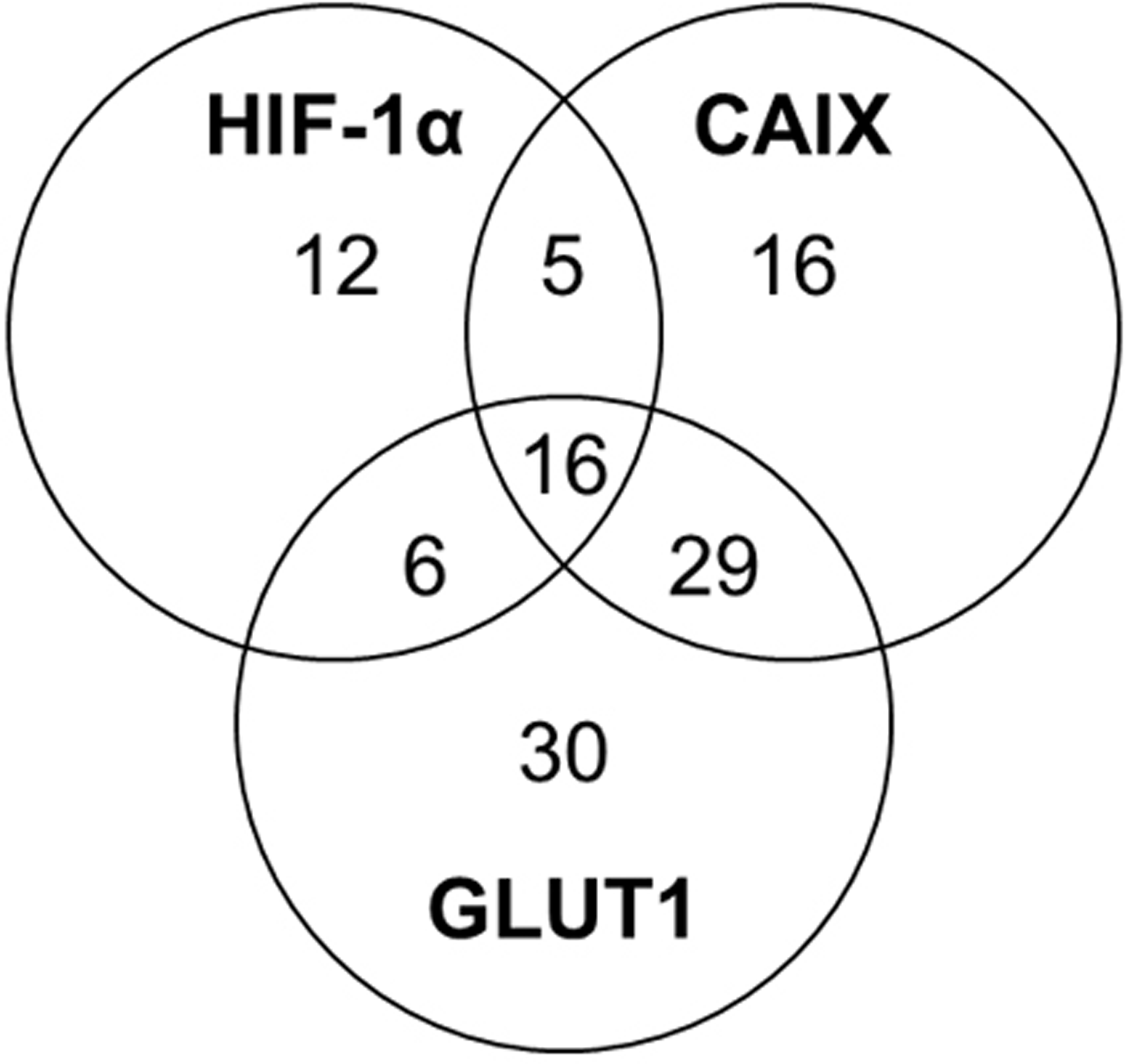

Supplement: Supplementary Figure 4 [file bjc2017430x4.tif]

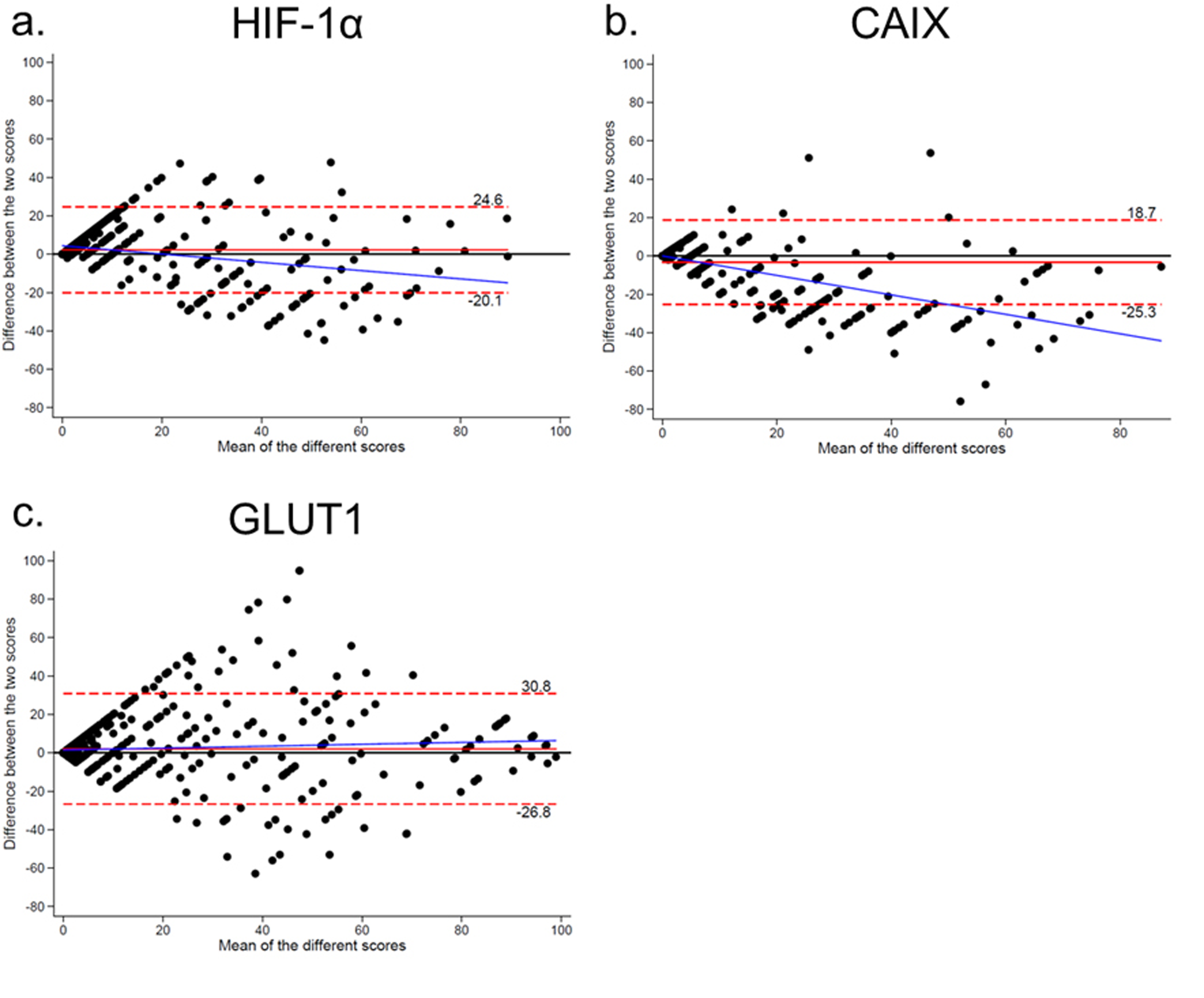

Supplement: Supplementary Figure 5 [file bjc2017430x5.tif]

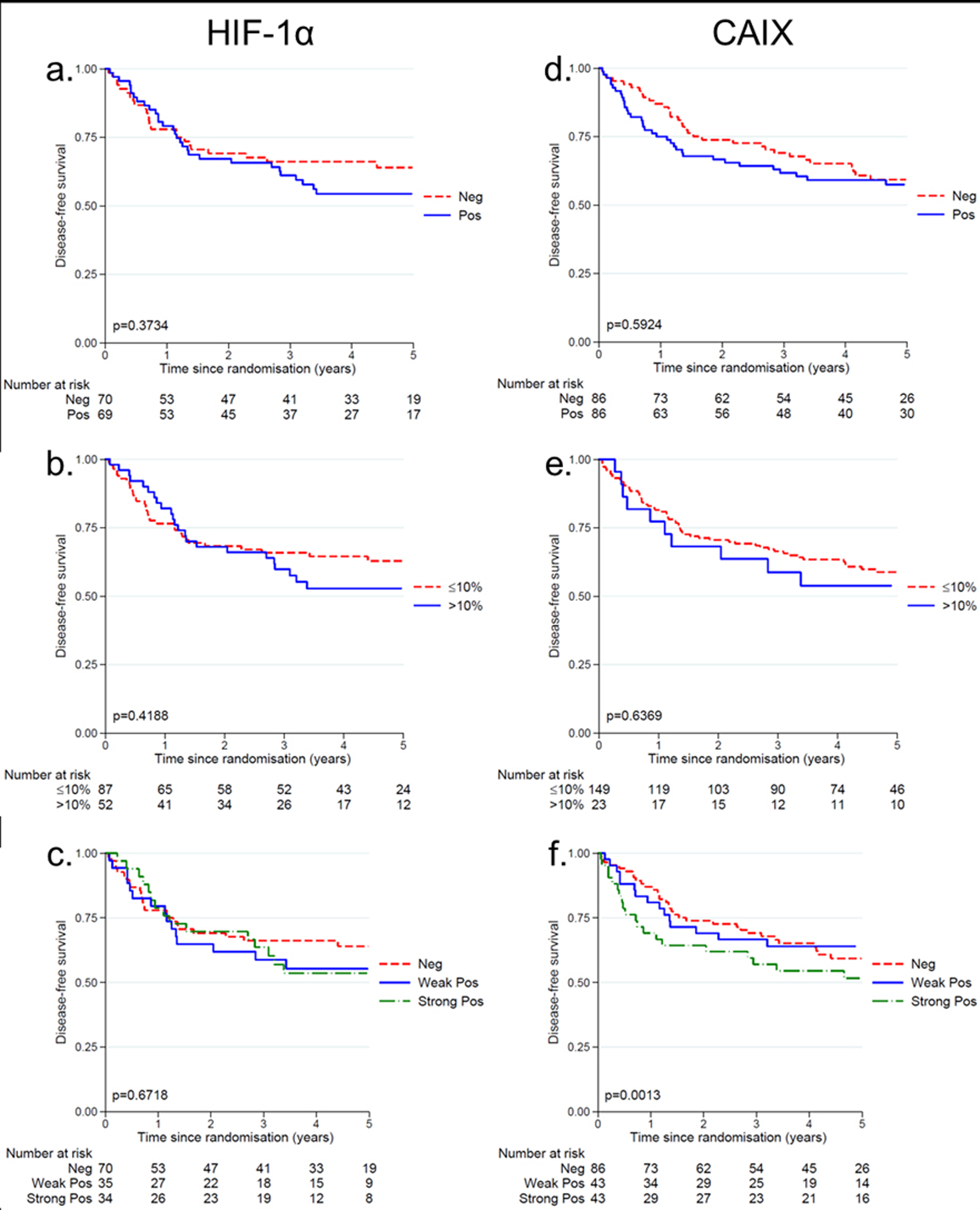

Supplement: Supplementary Figure 6 [file bjc2017430x6.tif]
